# Perioperative hemoglobin concentrations are associated with acute kidney injury after deceased donor liver transplantation

**DOI:** 10.3389/fmed.2026.1689545

**Published:** 2026-03-19

**Authors:** Paul Lichtenegger, Alexandra Graf, Judith Schiefer, Aylin Albrecht, Dagmar Kollmann, Gabriela A. Berlakovich, Peter Faybik, David M. Baron, Joanna Baron-Stefaniak

**Affiliations:** 1 Department of Anaesthesia, Intensive Care Medicine and Pain Medicine, Clinical Division of General Anaesthesia and Intensive Care Medicine, Medical University of Vienna, Vienna, Austria; 2 Institute of Medical Statistics, Centre for Medical Data Science, Medical University of Vienna, Vienna, Austria; 3 Division of Transplantation, Department of Surgery, Medical University of Vienna, Vienna, Austria; 4 Retired Head of Division of Transplantation, Department of Surgery, Medical University of Vienna, Vienna, Austria

**Keywords:** anemia, dialysis, kidney failure, morbidity, orthotopic liver transplantation, renal failure, renal replacement therapy, survival

## Abstract

**Background:** Acute kidney injury (AKI) is common after orthotopic liver transplantation (OLT). In major surgery, anemia has been associated with postoperative AKI. Although patients undergoing OLT frequently suffer from anemia, its impact on postoperative AKI remains unclear. We performed a single-center retrospective study including 724 patients undergoing their first OLT between January 2004 and December 2019 at the Medical University of Vienna. We evaluated whether perioperative hemoglobin concentrations were associated with postoperative AKI, renal replacement therapy, or mortality. Preoperative hemoglobin concentrations were 10.9 ( ± 2.1) g/dL in patients with AKI after OLT and 11.5 ( ± 2.1) g/dL in those without. Higher preoperative hemoglobin concentrations were associated with a lower probability of AKI (OR 0.847; *P* < 0.001) and a decreased probability of developing a higher AKI stage (OR: 0.895; *P* = 0.002). Stage 3 AKI was associated with increased 1-year (OR: 1.909; *P* < 0.001) and overall mortality (HR: 1.420; *P* = 0.037). Higher nadir hemoglobin concentrations within 48 h after OLT were associated with a lower probability of AKI (OR: 0.806; *P* = 0.033) and a decreased probability of developing a higher AKI stage (OR: 0.782; *P* < 0.001). In conclusion, higher perioperative hemoglobin concentrations were associated with a lower probability of AKI after OLT. Severe AKI was associated with an increased mortality.

## Introduction

Acute kidney injury (AKI) commonly occurs after major surgery (1–7). Patients undergoing orthotopic liver transplantation (OLT) are especially at high risk of developing postoperative AKI. Depending on the definition used, the incidence of AKI after OLT ranges from 43 to 78% (8–12). Moreover, up to 39% of patients require renal replacement therapy (RRT) after OLT (9, 13, 14). Both AKI and the requirement of RRT after OLT have a negative impact on patient outcome (9, 15).

Approximately 30% of patients scheduled for major surgery suffer from preoperative anemia, increasing the risk of perioperative morbidity and mortality (7, 16–19). In particular, preoperative anemia is associated with postoperative AKI and RRT in patients undergoing major cardiac and non-cardiac surgery (2–4, 7). Anemia occurs in up to 75% of patients requiring OLT (20–22), but its impact on outcome in this patient population remains controversial (9, 11, 23). Transfusion of packed red blood cells, which is frequently used to correct anemia, has been associated with adverse outcome and the development of postoperative AKI in patients undergoing OLT (23–27). However, it remains unclear whether the presence of perioperative anemia contributes to the development of postoperative AKI in patients undergoing OLT.

Based on our previous study (15), we focused our analyses on the association of perioperative hemoglobin concentrations with postoperative AKI in an extended dataset. The primary aim of this study was to evaluate whether patients with higher preoperative hemoglobin concentrations were at lower risk to develop AKI and require RRT after OLT. Secondarily, we assessed whether postoperative AKI was associated with a worse outcome following OLT. In addition, we assessed a potential association of early postoperative hemoglobin concentrations with AKI and outcome.

## Materials and methods

This single center retrospective study was performed at the Medical University of Vienna in accordance with ethical standards laid down in the declaration of Helsinki. The local ethics committee approved the study (EK 2347/2016) and the respective data protection plan. We evaluated all patients undergoing their first OLT between January 2004 and December 2019 for inclusion. Patients younger than 18 years and patients undergoing combined liver-lung or combined liver-kidney transplantation were excluded from analyses. We used the mortality registry released by “Statistics Austria” in 2020 to assess the date of death of deceased patients. Findings are presented in accordance with the STROBE guidelines (28).

### Study endpoints

The primary endpoint was the development of postoperative AKI within the first week after OLT. The secondary endpoints were the requirement of RRT, 1-year mortality, and overall mortality.

### Assessment of serum parameters

We assessed preoperative serum parameters within 24 h prior to OLT. Furthermore, we evaluated serum parameters for each postoperative day (POD) from standard blood analyses performed daily at 6 a.m. for 1 week following OLT. In addition to hemoglobin concentrations on POD 1 and 2, we evaluated and recorded the nadir hemoglobin concentrations within 48 h after OLT. Institutional transfusion thresholds were not standardized and could not be reliably determined retrospectively. Transfusion decisions were left to the discretion of the attending physician and were likely subject to temporal variation over the study period.

### Definition of acute kidney injury

We defined AKI using the serum creatinine and RRT criteria of the KDIGO classification (29). We did not use urine output for AKI classification. Baseline serum creatinine concentrations were assessed within 24 h prior to OLT. In order to adhere to the KDIGO criteria, we characterized patients who remained at AKI stage 0 within the first postoperative week as non-AKI patients and those who developed AKI stage 1, 2, or 3 as AKI patients. Furthermore, we categorized patients according to their highest individual AKI stage within the first week after OLT. According to KDIGO criteria, we classified all patients requiring RRT as AKI stage 3 regardless of serum creatinine concentrations. Additionally, we grouped patients by whether they required RRT within 1 week following OLT.

### Definitions of postoperative surgical complications and early allograft dysfunction

All complications requiring surgical revision within 4 weeks after OLT were regarded as postoperative surgical complications. Early allograft dysfunction was defined as the presence of one of the following characteristics: serum bilirubin concentrations ≥ 10 mg/dL on day 7 after OLT, INR ≥ 1.6 on day 7 after OLT, or serum aminotransferase (alanine aminotransferase, ALT, or aspartate aminotransferase, AST) concentrations > 2,000 IU/mL within the first week after OLT (30).

### Statistical analyses

First, we calculated descriptive statistics for continuous (mean ± standard deviation) and categorical (absolute and percentage values) variables. Next, we performed logistic or cox-regression analyses to investigate the association between various perioperative patient characteristics and the primary and secondary endpoints. For the endpoints AKI and RRT within 1 week, the following perioperative characteristics were included in the regression models: preoperative hemoglobin concentration (as continuous variable), estimated glomerular filtration rate according to the CKD-EPI formula (31), age, gender, body mass index (BMI), model for end-stage liver disease (MELD) score, coronary heart disease, chronic obstructive pulmonary disease, diabetes mellitus, cold ischemia time, warm ischemia time, intraoperative transfusion of packed red blood cells (PRBC), fresh frozen plasma (FFP), and platelets, immunosuppressive regime, as well as for POD 1 and 2 AST, ALT, bilirubin, c-reactive protein concentrations, and prothrombin time. For the endpoints 1-year mortality and overall mortality, we additionally included the postoperative confounders postoperative surgical complications, AKI (yes vs. no; as well as stages 0, 1, 2, and 3), and RRT within 1 week after OLT in the regression models. Moreover, we included early allograft dysfunction instead of AST, ALT, bilirubin, and prothrombin time in the mortality models.

In detail, the primary objective, i.e., the association of preoperative hemoglobin concentrations with the development of AKI (yes vs. no), was investigated by first performing univariable logistic regression followed by multivariable logistic regression with backward selection including all variables which were significant in the univariable models (*P* < 0.05). Only including significant parameters in the multivariable models was performed to reduce parameters included in the multivariable model. Backward selection was used to reduce potential overfitting or multicollinearity while starting from a clinically informed full model. This approach may retain potential key clinical predictors in the model and does not exclude them a priori. In addition, we performed univariable and multivariable ordered logistic regression models with backward selection to evaluate the association of preoperative hemoglobin concentrations with AKI stages 0, 1, 2, and 3. To investigate the secondary objective, namely the association of AKI with mortality after OLT, we performed univariable and multivariable cox or logistic regression analyses for overall and 1-year mortality.

Furthermore, we investigated the association of early postoperative hemoglobin concentrations with the development of AKI by repeating the above-mentioned univariable and multivariable regression analyses. Hemoglobin concentrations at POD 1, POD 2, and the nadir hemoglobin concentrations within 48 h after OLT were added to the models. Hemoglobin concentrations on POD 2 cannot be a risk factor for the development of AKI on POD 1. Thus, patients developing their highest individual AKI stage on POD 1 were excluded from all analyses using hemoglobin concentrations at POD 2 and nadir hemoglobin concentrations within 48 h after OLT.

In 2014, a new data management system was implemented at our center, which allowed us to obtain detailed timing of RRT initiation, PRBC and FFP transfusions, and albumin infusions. Hence, we investigated the association between hemoglobin concentrations and time to AKI onset in a subgroup of patients transplanted between January 2014 and December 2019. In this patient population, we performed additional cox-regression models including preoperative variables and immunosuppressive regime as mentioned above. In addition, we analyzed temporal associations within a cohort undergoing marked postoperative physiological changes, using time-dependent variables defined as follows: (i) hemoglobin, AST, ALT, bilirubin, c-reactive protein concentrations, and prothrombin time on the day of AKI diagnosis, (ii) hemoglobin concentrations on the day before AKI diagnosis, (iii) difference between preoperative hemoglobin concentrations and those on the day of AKI diagnosis, (iv) difference between hemoglobin concentrations on the day of AKI diagnosis and those the day before, (v) PRBC transfusions on the day of AKI diagnosis, (vi) FFP transfusions on the day of AKI diagnosis, and (vii) albumin infusions on the day of AKI diagnosis. In detail, we first performed univariable cox regression models with AKI as an event for these time-dependent covariates. Next, a multivariable model with backward selection was performed including all variables which were significant in the univariable models (*P* < 0.05). Time-dependent regression models were performed independently for each AKI stage (time to AKI stage ≥ 1, time to AKI stage ≥ 2, and time to AKI stage 3). Multicollinearity was evaluated using variance inflation factors.

## Results

Of 788 patients who underwent their first OLT between January 2004 and December 2019, 64 patients met the exclusion criteria [age < 18 (*n* = 34); combined liver-kidney transplantation (*n* = 24); combined liver-lung transplantation (*n* = 6)]. Thus, we included 724 patients in our final analyses. Detailed demographic and perioperative characteristics are shown in Table 1. The main indications for OLT were alcoholic liver cirrhosis (28.6%), hepatocellular carcinoma (24.4%), and hepatitis C virus cirrhosis (13.3%) (Supplementary Table 1). All OLTs were performed using the bi-caval clamping technique without the routine use of veno-venous bypass.

**TABLE 1:** Demographic data and perioperative characteristics of the study population.

Parameters	All patients (*n* = 724)	No AKI (*n* = 233)	AKI (*n* = 491)	*P*-value
Preoperative characteristics				
Age, years	54 ± 10	53 ± 11	54 ± 10	0.076
Male sex	532 (73.5)	152 (65.2)	380 (77.4)	**< 0.001**
Body mass index, kg m^–2^	26.2 ± 4.5	24.8 ± 4.0	26.8 ± 4.6	**< 0.001**
MELD score	19 ± 7	18 ± 7	20 ± 7	**< 0.001**
Coronary heart disease	25 (3.5)	7 (3)	18 (3.7)	0.829
Chronic obstructive pulmonary disease	43 (5.9)	13 (5.6)	30 (6.1)	0.932
Diabetes mellitus	163 (22.5)	43 (18.5)	120 (24.4)	0.099
Estimated glomerular filtration rate	81 ± 28	80 ± 29	81 ± 29	0.542
Preoperative hospitalization	89 (12.3)	20 (8.6)	69 (14.1)	**0.049**
Hemoglobin concentrations (g dL^–1^)				
Preoperative	11.1 ± 2.1	11.5 ± 2.1	10.9 ± 2.1	**0.001**
POD 1	9.5 ± 1.5	9.7 ± 1.6	9.4 ± 1.5	**0.018**
POD 2	9.2 ± 1.3	9.4 ± 1.3	9.1 ± 1.3	**0.004**
Nadir within 48 h	8.6 ± 1.3	8.9 ± 1.4	8.4 ± 1.2	**< 0.001**
Intraoperative characteristics				
Cold ischemia time, min	472 ± 140	452 ± 143	482 ± 138	**0.014**
Warm ischemia time, min	79 ± 19	74 ± 18	80 ± 20	**< 0.001**
PRBC transfusions, n	4 ± 6	3 ± 5	5 ± 6	**0.005**
FFP transfusions, n	9 ± 8	7 ± 8	10 ± 7	**0.001**
Platelet transfusions, n	1 ± 1	0 ± 1	1 ± 1	**< 0.001**
Postoperative characteristics				
Immunosuppression with Fk506, n (%)	264 (36.5)	76 (32.6)	188 (38.3)	0.139
Surgical complications, n (%)	200 (27.6)	44 (18.9)	156 (31.8)	**< 0.001**
Early allograft dysfunction, n (%)	192 (26.5)	33 (14.2)	159 (32.4)	**< 0.001**

Data are shown in n (%), mean ± standard deviation. *P*-values are shown for chi-square/fisher exact tests (categorical variables) and two-sample *t*-tests (continuous variables); significant values are shown in bold. Abbreviations: AKI, acute kidney injury; FFP, fresh frozen plasma; MELD, model for end-stage liver disease; POD, postoperative day; PRBC, packed red blood cell.

### Incidence of AKI after OLT

Of the 724 included patients, 491 patients (68%) developed AKI within the first postoperative week (201/491 (41%) AKI stage 1, 88/491 (18%) AKI stage 2, 202/491 (41%) AKI stage 3). In total, 162/724 patients (22%) required renal replacement therapy (RRT) within the first postoperative week.

### Preoperative hemoglobin concentrations and AKI after OLT

**FIGURE 1:**
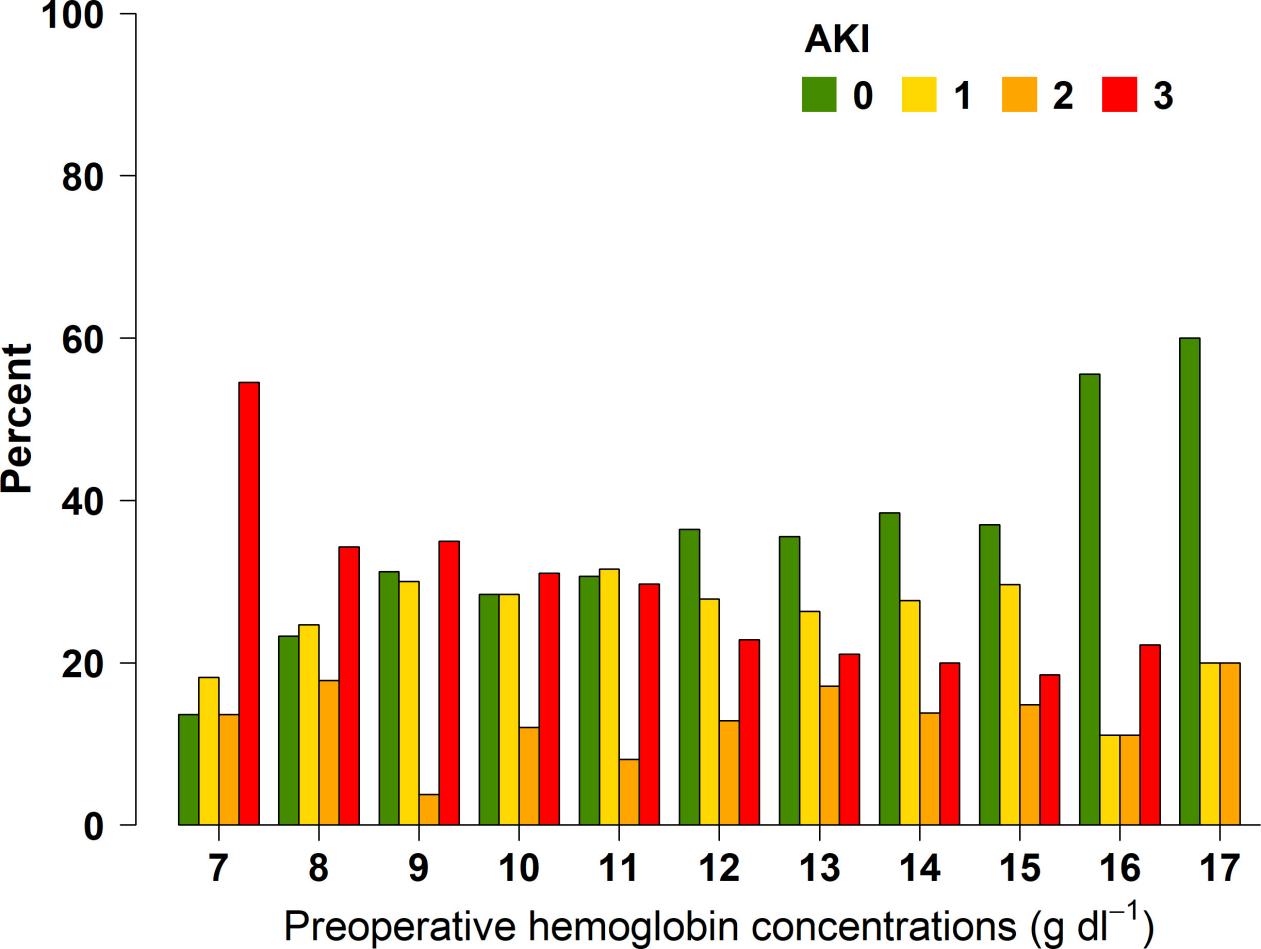
Percentage of patients with their respective postoperative AKI stages according to preoperative hemoglobin concentrations. Note that percentages reflect the proportions of patients within each hemoglobin category.

Average preoperative hemoglobin concentrations were 10.9 ( ± 2.1) g/dL in patients who developed AKI within the first week after OLT and 11.5 ( ± 2.1) g/dL in those who did not (*P* = 0.001) (Table 1). Although this difference is statistically significant, its clinical relevance remains uncertain. Multivariable logistic regression analysis revealed that higher preoperative hemoglobin concentrations were significantly associated with a lower probability for the development of postoperative AKI (OR 0.847; 95% CI: 0.773–0.928; *P* < 0.001) (Table 2; Supplementary Table 2). Additionally, multivariable ordered logistic regression analysis showed that the probability to develop a higher AKI stage decreased with higher preoperative hemoglobin concentrations (OR: 0.895; 95% CI: 0.826–0.970; *P* = 0.002) (Figure 1; Table 2; Supplementary Table 2). Furthermore, in our time-dependent analyses, higher preoperative hemoglobin concentrations were significantly associated with a lower probability of postoperative AKI stage 3 in the univariable cox regression analysis (HR: 0.891; 95% CI: 0.807–0.985; *P* = 0.024) (Supplementary Table 3). However, this association did not remain significant in the multivariable model. Note that candidate covariates for multivariable models were first tested in univariable models and only significant ones entered multivariable models, see Statistical methods section and Supplementary Tables 2, 3. Similarly, preoperative hemoglobin concentrations were not significantly associated with the requirement of RRT within 1 week after OLT in the multivariable model (Table 2).

**TABLE 2:** Results of regression analyses on the influence of preoperative hemoglobin concentrations on AKI after orthotopic liver transplantation.

Endpoint parameters	Preoperative hemoglobin as influence factor					
	Univariable			Multivariable		
	OR	95% CI	*P*-value	OR	95% CI	*P*-value
AKI within 1 week of OLT						
log regression (yes vs. no)	0.883	0.820–0.952	**0.001**	0.847	0.773–0.928	**< 0.001**
ordered log regression (0, 1, 2, 3)	0.876	0.822–0.932	**< 0.001**	0.895	0.826–0.970	**0.002**
RRT within 1 week of OLT	0.834	0.765–0.910	**< 0.001**			n.s.

AKI, acute kidney injury; CI, confidence interval; log, logistic; n.s., not significant; OLT, orthotopic liver transplantation; OR, odds ratio; RRT, renal replacement therapy. Statistically significant values are shown in bold.

### AKI and mortality after OLT

**FIGURE 2:**
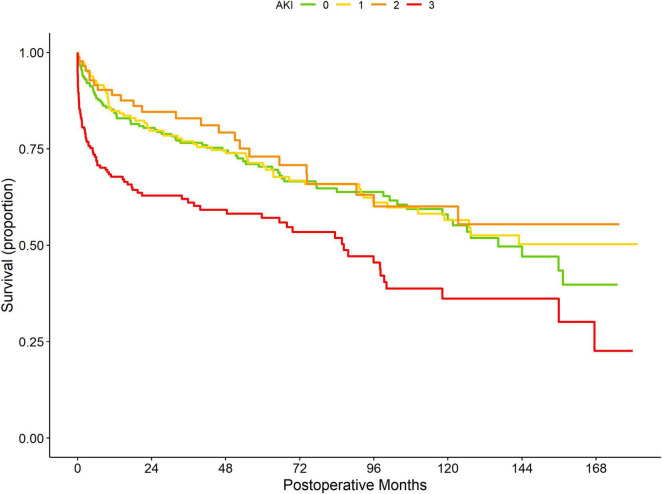
Postoperative unadjusted Kaplan-Meier curves according to AKI stages (stage 0 = green; stage 1 = yellow, stage 2 = orange, stage 3 = red).

Regression analyses showed no significant association between postoperative AKI (yes vs. no) and 1-year or overall mortality (Table 3). However, more detailed analyses revealed that AKI stage 3 was associated with a significantly increased 1-year mortality risk (OR: 1.909; 95% CI: 1.129–3.229; *P* < 0.001) (Table 3) and overall mortality risk (HR: 1.420; 95% CI: 1.021–1.975; *P* = 0.037) (Figure 2; Table 3). In contrast, preoperative hemoglobin concentrations did not show a significant association with 1-year mortality or overall mortality after OLT (Table 3).

**TABLE 3:** Results of regression analyses on the association of perioperative parameters with mortality after orthotopic liver transplantation.

	Univariable			Multivariable		
Parameters	OR/HR	95% CI	*P*-value	OR/HR	95% CI	*P*-value
Factors associated with 1-year mortality after OLT—logistic regression						
AKI (yes vs. no)	1.582	1.003–2.424	**0.035**			n.s.
AKI stage 1 vs. 0	0.988	0.574–1.700	0.964			n.s.
AKI stage 2 vs. 0	0.687	0.314–1.505	0.348			n.s.
AKI stage 3 vs. 0	2.936	1.820–4.737	**< 0.001**	1.909	1.129–3.229	**< 0.001**
PreOP Hb concentrations	1.022	0.933–1.120	0.641			
IntraOP PRBC transfusions	1.112	1.072–1.153	**< 0.001**	1.087	1.047–1.129	**< 0.001**
PostOP surgical complications	3.293	2.209–4.908	**< 0.001**	2.425	1.572–3.742	**< 0.001**
Factors associated with overall mortality after OLT—cox regression						
AKI (yes vs. no)	1.204	0.924–1.568	0.168			n.s.
AKI stage 1 vs. 0	0.923	0.663–1.284	0.633			n.s.
AKI stage 2 vs. 0	0.807	0.511–1.274	0.357			n.s.
AKI stage 3 vs. 0	1.847	1.365–2.499	**< 0.001**	1.420	1.021–1.975	**0.037**
PreOP Hb concentrations	1.046	0.985–1.110	0.140			
Age	1.025	1.012–1.039	**< 0.001**	1.023	1.009–1.037	**0.002**
Diabetes mellitus	1.395	1.058–1.840	**0.018**	1.333	1.002–1.774	**0.049**
IntraOP PRBC transfusions	1.068	1.049–1.087	**< 0.001**	1.054	1.030–1.079	**< 0.001**
PostOP surgical complications	1.755	1.357–2.270	**< 0.001**	1.413	1.072–1.863	**0.014**

AKI, acute kidney injury; CI, confidence interval; Hb, hemoglobin; HR, hazard ratio; IntraOP, intraoperative; n.s., not significant; OLT, orthotopic liver transplantation; OR, odds ratio; PostOP, postoperative; PRBC, packed red blood cell; PreOP, preoperative. Statistically significant values are shown in bold.

### Postoperative hemoglobin concentrations, AKI, and outcome after OLT

As hemoglobin concentrations on POD 2 cannot influence the development of AKI on POD 1, 202/724 (28%) patients reaching their highest AKI stage (AKI > 0) within the first day after surgery were excluded from the subsequent analysis. We observed that early postoperative hemoglobin concentrations at the analyzed time points were lower in AKI patients than in non-AKI patients (Table 1). However, the calculated difference between the hemoglobin concentrations of AKI and non-AKI patients did not exceed 0.5 g/dL at any analyzed time point and might be considered as not clinically relevant.

Multivariable logistic regression analysis revealed that higher nadir hemoglobin concentrations within 48 h after OLT were significantly associated with a lower probability for the development of postoperative AKI (OR: 0.806; 95% CI: 0.661–0.983; *P* = 0.033) (Table 4; Supplementary Table 2). In addition, multivariable ordered logistic regression analysis showed that the probability to develop a higher AKI stage decreased with higher nadir hemoglobin concentrations within 48 h after OLT (OR: 0.782; 95% CI: 0.661–0.921; *P* < 0.001) (Table 4; Supplementary Table 2). In contrast, postoperative hemoglobin concentrations were not significantly associated with 1-year or overall mortality at any analyzed time-point (Table 4).

**TABLE 4:** Results of regression analyses on the association of postoperative hemoglobin concentrations with the indicated outcome after orthotopic liver transplantation.

Parameters	Univariable			Multivariable		
	OR/HR	95% CI	*P*-value	OR/HR	95% CI	*P*-value
AKI (yes vs. no)—logistic regression						
Hb POD 1*	0.882	0.797–0.976	**0.015**			n.s.
Hb POD 2^†^	0.878	0.767–1.004	0.058			
Hb nadir POD 1, 2^†^	0.746	0.644–0.865	**< 0.001**	0.806	0.661–0.983	**0.033**
AKI (0, 1, 2, 3)—ordered logistic regression						
Hb POD 1*	0.898	0.822–0.980	**0.016**			n.s.
Hb POD 2^†^	0.891	0.785–1.010	0.073			
Hb nadir POD 1, 2^†^	0.741	0.644–0.850	**< 0.001**	0.782	0.661–0.921	**< 0.001**
One-year mortality—logistic regression						
Hb POD 1	0.892	0.787–1.012	0.076			
Hb POD 2	0.920	0.786–1.076	0.297			
Hb nadir POD 1, 2	0.886	0.753–1.042	0.143			
Overall mortality—cox regression						
Hb POD 1	0.944	0.872–1.021	0.15			
Hb POD 2	0.937	0.847–1.037	0.21			
Hb nadir POD 1, 2	0.917	0.828–1.017	0.101			

AKI, acute kidney injury; CI, confidence interval; Hb, hemoglobin concentrations; HR, hazard ratio; n.s., not significant; OR, odds ratio; POD, postoperative day.

*n = 724;

† *n* = 522. Statistically significant values are shown in bold.

### Perioperative hemoglobin concentrations and time to AKI onset

In time-dependent analyses within the subgroup of patients transplanted between January 2014 and December 2019, we assessed whether hemoglobin concentrations around the time of AKI diagnosis may be associated with the time to the onset of AKI. Neither hemoglobin concentrations on the same day, nor those 1 day before AKI diagnosis showed a significant association with the time to AKI onset. Furthermore, changes in hemoglobin concentrations were not significantly associated with the time to AKI onset in our time-dependent cox regression models (Supplementary Table 3).

Multivariable time-dependent analyses showed that the administration of PRBCs as well as AST, ALT, Bilirubin, and c-reactive protein concentrations on the day of AKI diagnosis remained significantly associated with an increased probability of an earlier development of AKI. Additionally, intraoperative FFP administration, BMI, and MELD score were significantly associated with an earlier development of AKI in multivariable analyses (Supplementary Table 3).

## Discussion

In this study, we investigated the association of perioperative hemoglobin concentrations with AKI and mortality after OLT. We report that higher preoperative hemoglobin concentrations were associated with a lower probability for the development of AKI following OLT in our patient population. Furthermore, the nadir hemoglobin level within 48 h after OLT was associated with an increased probability of developing AKI. Postoperative hemoglobin levels at or preceding AKI diagnosis and their temporal changes, however, were not significantly associated with the onset of AKI. Importantly, our results indicate that the development of severe AKI after OLT was significantly associated with increased 1-year mortality and overall mortality.

In our study population, 68% of patients developed AKI following OLT, corroborating previously published results (9–12, 15). This high incidence of AKI and its negative impact on postoperative outcome underlines the necessity to identify perioperative risk factors for AKI. Perioperative conditions, such as the severity of liver disease, severe intraoperative blood loss, early allograft dysfunction, or sepsis have already been identified as risk factors for AKI following OLT (23, 32, 33). However, it is challenging to modify these conditions to prevent AKI.

We evaluated anemia as a potential and modifiable risk factor for the development of AKI following OLT. Only few studies have included preoperative hemoglobin as a confounder in patients undergoing OLT (9, 11, 34, 35), yielding conflicting results. In a study including 116 patients undergoing OLT, Rahman et al. found no association of preoperative hemoglobin concentrations with early postoperative AKI (9). Similarly, Carrier et al. found no association of preoperative hemoglobin concentrations with AKI after OLT (11). Importantly, patients with hemoglobin concentrations above 8.5 g/dl and a normal renal function underwent phlebotomy (7–10 mL/kg) prior to transplantation (11). This procedure may limit the validity of the findings since hemoglobin concentrations were artificially lowered after the assessment of baseline hemoglobin concentrations immediately prior to transplantation. In contrast, Kim et al. reported an association between lower preoperative hemoglobin levels and AKI after living donor liver transplantation (34), whereas Erdost et al. observed such an association only when AKI was defined using RIFLE criteria but not KDIGO criteria (35).

There are several differences between our study and previously published investigations. Whereas Kim et al. excluded patients receiving organs from deceased donors and patients with an estimated preoperative glomerular filtration rate of less than 60 mL/min/1.73 m^2^ (34), we included all patients following deceased donor OLT, irrespective of their preoperative kidney function. The inclusion of patients with preoperative renal dysfunction allowed for a more comprehensive characterization of the study population. Notably, we observed no association between preoperative estimated glomerular filtration rate and the development of postoperative AKI. In contrast to Erdost et al. (35), hemoglobin concentrations were analyzed as a continuous variable to provide a more nuanced assessment of their impact on postoperative outcomes. This approach is particularly relevant in patients with end-stage liver disease, who frequently suffer anemia. Furthermore, AKI was assessed exclusively using KDIGO criteria. The incidence of AKI reported by Erdost et al. (14%) was relatively low compared with our findings and those of previous investigations (9–12, 15). Potential explanations for this lower incidence might be split liver transplantation from living donor grafts, the use of the piggyback technique for vena cava reconstruction during OLT, or the relatively low age of transplant recipients (36, 37).

Preoperative anemia is known to impair outcome after major cardiac and non-cardiac surgery (7, 16, 18, 19). Although anemia is common in patients suffering from end-stage liver disease, its impact on mortality after OLT remains controversial. Badawy et al. reported that preoperative hemoglobin concentrations below 10 g/dl were associated with greater mortality within 90 days after liver transplantation (22). However, other studies did not support these results (15, 20). Collas et al. found no association of anemia with mortality following OLT (20). In addition, the results of our previous study show that preoperative hemoglobin concentrations were not associated with increased mortality after OLT (15). Consistent with our previous findings, we did not observe any significant association between preoperative hemoglobin concentrations and mortality in the current investigation. Nevertheless, our results suggest that patients who develop severe AKI after OLT may be at risk for increased 1-year mortality and overall mortality.

In our study, increased mortality was observed only in patients who developed severe AKI (stage 3). This contrasts with findings from other surgical populations, in which AKI has been linked to increased mortality regardless of severity. Kork et al. reported that even subclinical AKI was associated with increased mortality in non-cardiac surgical patients in a study including 39,369 postoperative patients (5). Similarly, in a study on 2,322 patients, Haase et al. demonstrated that even subclinical AKI was associated with worse outcome in cardiorenal syndrome type 1 (38). In patients suffering from end-stage liver disease, renal function tends to be overestimated due to falsely low serum creatinine concentrations. Contrarily, post-liver transplant renal function tends to be underestimated due to a drug induced reduction in tubular secretion of creatinine, potentially leading to overdiagnosis of AKI after OLT (39). This may partially explain our findings why only severe AKI was associated with increased mortality.

In addition to preoperative hemoglobin concentrations, we investigated the association of postoperative hemoglobin concentrations with AKI. A decrement in perioperative hemoglobin concentrations has been associated with the development of AKI in patients undergoing non-cardiac surgery (3). In contrast, no significant association between postoperative hemoglobin concentrations and AKI was reported in a study comparing RIFLE, AKIN, and KDIGO criteria for AKI following OLT (35). Concordantly, we observed that hemoglobin concentrations neither on POD 1 nor on POD 2 were significantly associated with AKI. However, nadir hemoglobin concentrations within 48 h of OLT were significantly associated with the development of AKI in our multivariable analysis. Our findings agree with results from previous studies describing that lower postoperative nadir hemoglobin concentrations were associated with inferior outcome after cardiac and abdominal surgery (40, 41).

The most common treatment of anemia in the perioperative setting is transfusion of PRBCs. In our cohort, transfusion of PRBCs during OLT was associated with higher mortality, a finding consistent with previous reports in patients undergoing OLT and other major surgical procedures (24, 25, 42–44). Notably, the relatively low number of transfused units constrains the assessment of their potential effect on outcomes. Furthermore, transfusion of PRBCs on the day of AKI diagnosis was significantly associated with an earlier onset of AKI. These observations underscore the close relationship between transfusion practices and adverse postoperative outcomes and suggest that PRBC transfusions may act as an important confounding factor in the association between hemoglobin concentrations, AKI, and mortality.

Patient blood management is a multimodal concept designed to reduce perioperative transfusion requirements and improve postoperative patient outcome. While the intra- and postoperative approaches focus on minimizing blood loss and increasing anemia tolerance, the preoperative approach aims to optimize erythrocyte mass before surgery (45). Several studies on patients undergoing oncologic, orthopedic, and abdominal surgery have demonstrated that treating anemia prior to surgery resulted in reduced transfusion requirements and improved perioperative outcome (46–49). While it has not been investigated thus far, it could be expected that patients awaiting OLT might benefit from preoperative treatment of anemia. The main causes of reduced hemoglobin concentrations in end-stage liver disease include gastrointestinal bleeding, malnutrition, iron deficiency, folate deficiency, vitamin B12 deficiency, and myelosuppression (21, 50–53). Our study provides insight that may inform future investigations on whether preoperative management of anemia with iron infusions, substitution of folate and vitamin B12, and erythropoiesis-stimulating agents could be associated with improved postoperative outcomes after OLT.

A strength of our study is the large number of included patients. Furthermore, data on pre- and postoperative hemoglobin concentrations allowed us to perform temporally detailed perioperative analyses. To our knowledge, this is the first study providing detailed analyses on the association of postoperative hemoglobin concentrations with outcomes after OLT. Our study has several limitations. One limitation is that we could only use the serum creatinine criteria of the KDIGO-classification to assess AKI stages, as it was not possible to retrospectively extract hourly urine outputs, potentially leading to misclassification of AKI in some patients. In addition, all patients receiving RRT were automatically classified as AKI stage III leading to a potential overrepresentation of this category. Furthermore, we were unable to determine the causes of anemia in our patient population due to missing data. Moreover, PRBC transfusions and varying transfusion triggers may have substantially influenced hemoglobin concentrations in some patients, potentially introducing bias into the analyses. Blood transfusion was accounted for in the multivariable models, however no further sensitivity analyses were performed but may be valuable. In addition, some effect sizes observed reached statistical significance but were small in magnitude and therefore may not be clinically significant. Another limitation of our study is its retrospective and monocentric design, which allows only to conclude associations rather than causal relationships and might limit the generalizability of our results. This is further reflected by the exclusion of extremely frail patients from OLT at our institution, whereas such patients may be transplanted at other centers. Moreover, the observation period in this study spans more than 15 years, in which surgical procedures, medical treatments, and transfusion practices may have changed substantially.

## Conclusion

In conclusion, this study suggests that higher perioperative hemoglobin concentrations are significantly associated with a lower probability for the development of AKI following OLT. Importantly, we observed that severe AKI following OLT was significantly associated with increased 1-year and overall mortality.

## Data Availability

The datasets presented in this article are not readily available because of data protection. Data that support the findings of this study are available upon reasonable request from the corresponding author. Requests to access the datasets should be directed to alexandra.graf@meduniwien.ac.at.

## References

[1] Biteker M Dayan A Tekkeşin Aİ Can MM Taycİ İ İlhan E et al. Incidence, risk factors, and outcomes of perioperative acute kidney injury in noncardiac and nonvascular surgery. *Am J Surg.* (2014) 207:53–9. 10.1016/j.amjsurg.2013.04.006 24050540

[2] Karkouti K Yip P Chan C Chawla L Rao V. Pre-operative anaemia, intra-operative hepcidin concentration and acute kidney injury after cardiac surgery: a retrospective observational study. *Anaesthesia.* (2018) 73:1097–102. 10.1111/anae.14274 29529338

[3] Walsh M Garg AX Devereaux PJ Argalious M Honar H Sessler DI. The association between perioperative hemoglobin and acute kidney injury in patients having noncardiac surgery. *Anesth Analg.* (2013) 117:924–31. 10.1213/ANE.0b013e3182a1ec84 24023017

[4] Haase M Bellomo R Story D Letis A Klemz K Matalanis G et al. Effect of mean arterial pressure, haemoglobin and blood transfusion during cardiopulmonary bypass on post-operative acute kidney injury. *Nephrol Dial Transpl.* (2012) 27:153–60. 10.1093/ndt/gfr275 21677302

[5] Kork F Balzer F Spies CD Wernecke KD Ginde AA Jankowski J et al. Minor postoperative increases of creatinine are associated with higher mortality and longer hospital length of stay in surgical patients. *Anesthesiology.* (2015) 123:1301–11. 10.1097/ALN.0000000000000891 26492475 PMC4679549

[6] Hansen MK Gammelager H Mikkelsen MM Hjortdal VE Layton JB Johnsen SP et al. Post-operative acute kidney injury and five-year risk of death, myocardial infarction, and stroke among elective cardiac surgical patients: a cohort study. *Crit Care.* (2013) 17:R292. 10.1186/cc13158 24330762 PMC4057271

[7] Warner MA Hanson AC Schulte PJ Sanz JR Smith MM Kauss ML et al. Preoperative anemia and postoperative outcomes in cardiac surgery: a mediation analysis evaluating intraoperative transfusion exposures. *Anesth Analg.* (2024) 138:728–37. 10.1213/ANE.0000000000006765 38335136 PMC10949062

[8] Berkowitz RJ Engoren MC Mentz G Sharma P Kumar SS Davis R et al. Intraoperative risk factors of acute kidney injury after liver transplantation. *Liver Transpl.* (2022) 28:1207–23. 10.1002/lt.26417 35100664 PMC9321139

[9] Rahman S Davidson BR Mallett SV. Early acute kidney injury after liver transplantation: Predisposing factors and clinical implications. *World J Hepatol.* (2017) 9:823–32. 10.4254/wjh.v9.i18.823 28706581 PMC5491405

[10] Hilmi IA Damian D Al-Khafaji A Planinsic R Boucek C Sakai T et al. Acute kidney injury following orthotopic liver transplantation: incidence, risk factors, and effects on patient and graft outcomes. *Br J Anaesth.* (2015) 114:919–26. 10.1093/bja/aeu556 25673576

[11] Carrier FM Chassé M Sylvestre MP Girard M Legendre-Courville L Massicotte L et al. Effects of intraoperative fluid balance during liver transplantation on postoperative acute kidney injury: an observational cohort study. *Transplantation.* (2020) 104:1419–28. 10.1097/TP.0000000000002998 31644490

[12] Barri YM Sanchez EQ Jennings LW Melton LB Hays S Levy MF et al. Acute kidney injury following liver transplantation: definition and outcome. *Liver Transpl.* (2009) 15:475–83. 10.1002/lt.21682 19399734

[13] Zand MS Orloff MS Abt P Patel S Tsoulfas G Kashyap R et al. High mortality in orthotopic liver transplant recipients who require hemodialysis. *Clin Transpl.* (2011) 25:213–21. 10.1111/j.1399-0012.2010.01238.x 20331690

[14] Stefaniak J Schiefer J Miller EJ Krenn CG Baron DM Faybik P. Macrophage migration inhibitory factor as a potential predictor for requirement of renal replacement therapy after orthotopic liver transplantation. *Liver Transpl.* (2015) 21:662–9. 10.1002/lt.24103 25762421

[15] Lichtenegger P Schiefer J Graf A Berlakovich G Faybik P Baron DM et al. The association of pre-operative anaemia with survival after orthotopic liver transplantation. *Anaesthesia.* (2020) 75:472–8. 10.1111/anae.14918 31701527 PMC7078747

[16] Baron DM Hochrieser H Posch M Metnitz B Rhodes A Moreno RP et al. Preoperative anaemia is associated with poor clinical outcome in non-cardiac surgery patients. *Br J Anaesth.* (2014) 113:416–23. 10.1093/bja/aeu098 24829444

[17] Muñoz M Laso-Morales MJ Gómez-Ramírez S Cadellas M Núñez-Matas MJ García-Erce JA. Pre-operative haemoglobin levels and iron status in a large multicentre cohort of patients undergoing major elective surgery. *Anaesthesia.* (2017) 72:826–34. 10.1111/anae.13840 28382661

[18] Musallam KM Tamim HM Richards T Spahn DR Rosendaal FR Habbal A et al. Preoperative anaemia and postoperative outcomes in non-cardiac surgery: a retrospective cohort study. *Lancet.* (2011) 378:1396–407. 10.1016/S0140-673661381-0 21982521

[19] LaPar DJ Hawkins RB McMurry TL Isbell JM Rich JB Speir AM et al. Preoperative anemia versus blood transfusion: which is the culprit for worse outcomes in cardiac surgery? *J Thorac Cardiovasc Surg.* (2018) 156:66.e–74.e. 10.1016/j.jtcvs.2018.03.109 29706372 PMC6093299

[20] Collas O Robertson FP Fuller BJ Davidson BR. Anaemia in patients with chronic liver disease and its association with morbidity and mortality following liver transplantation. *Int J Surg.* (2018) 53:48–52. 10.1016/j.ijsu.2018.02.053 29499362

[21] Gonzalez-Casas R Jones EA Moreno-Otero R. Spectrum of anemia associated with chronic liver disease. *World J Gastroenterol.* (2009) 15:4653–8. 10.3748/wjg.15.4653 19787828 PMC2754513

[22] Badawy A Kaido T Hammad A Yagi S Kamo N Yoshizawa A et al. The impact of preoperative hemoglobin level on the short-term outcomes after living donor liver transplantation. *World J Surg.* (2018) 42:4081–9. 10.1007/s00268-018-4696-5 29882099

[23] Chen J Singhapricha T Hu KQ Hong JC Steadman RH Busuttil RW et al. Postliver transplant acute renal injury and failure by the RIFLE criteria in patients with normal pretransplant serum creatinine concentrations: a matched study. *Transplantation.* (2011) 91:348–53. 10.1097/TP.0b013e31820437da 21127462

[24] de Boer MT Christensen MC Asmussen M van der Hilst CS Hendriks HGD Slooff MJH et al. The impact of intraoperative transfusion of platelets and red blood cells on survival after liver transplantation. *Anesth Analg.* (2008) 106:32–44. 10.1213/01.ane.0000289638.26666.ed 18165548

[25] Ramos E Dalmau A Sabate A Lama C Llado L Figueras J et al. Intraoperative red blood cell transfusion in liver transplantation: Influence on patient outcome, prediction of requirements, and measures to reduce them. *Liver Transplantation.* (2003) 9:1320–7. 10.1016/jlts.2003.50204 14625833

[26] Park MH Shim HS Kim WH Kim HJ Kim DJ Lee SH et al. Clinical Risk Scoring Models for Prediction of Acute Kidney Injury after Living Donor Liver Transplantation: A Retrospective Observational Study. *PLoS One.* (2015) 10:e0136230. 10.1371/journal.pone.0136230 26302370 PMC4547769

[27] Seong H Jang Y Ko E Lee J Kim T Lim CH et al. Impact of preoperative red blood cell transfusion on long-term mortality of liver transplantation: A retrospective cohort study. *Medicine (Baltimore).* (2023) 102:e34914. 10.1097/MD.0000000000034914 37713857 PMC10508566

[28] von Elm E Altman DG Egger M Pocock SJ Gøtzsche PC Vandenbroucke JP. The Strengthening the Reporting of Observational Studies in Epidemiology (STROBE) statement: guidelines for reporting observational studies. *Journal of Clinical Epidemiology.* (2008) 61:344–9. 10.1136/bmj.39335.541782.AD 18313558

[29] Kidney Disease: Improving Global Outcomes (Kdigo) Acute Kidney Injury Work Group. KDIGO Clinical Practice Guideline for Acute Kidney Injury. *Kidney International Supplements* (2012) 2:1. 10.1038/kisup.2012.2 25028631 PMC4089660

[30] Olthoff KM Kulik L Samstein B Kaminski M Abecassis M Emond J et al. Validation of a current definition of early allograft dysfunction in liver transplant recipients and analysis of risk factors. *Liver Transpl.* (2010) 16:943–9. 10.1002/lt.22091 20677285

[31] Levey AS Stevens LA Schmid CH Zhang YL Castro AF Feldman HI et al. A new equation to estimate glomerular filtration rate. *Ann Intern Med.* (2009) 150:604–12. 10.7326/0003-4819-150-9-200905050-00006 19414839 PMC2763564

[32] Romano TG Schmidtbauer I Silva FM de Q Pompilio CE D’Albuquerque LAC Macedo E. Role of MELD score and serum creatinine as prognostic tools for the development of acute kidney injury after liver transplantation. *PLoS One.* (2013) 8:e64089. 10.1371/journal.pone.0064089 23717537 PMC3662723

[33] Cabezuelo JB Ramírez P Ríos A Acosta F Torres D Sansano T et al. Risk factors of acute renal failure after liver transplantation. *Kidney Int.* (2006) 69:1073–80. 10.1038/sj.ki.5000216 16528257

[34] Kim WH Lee HC Lim L Ryu HG Jung CW. Intraoperative oliguria with decreased SvO2 predicts acute kidney injury after living donor liver transplantation. *J Clin Med.* (2018) 8:E29. 10.3390/jcm8010029 30597881 PMC6351957

[35] Erdost HA Ozkardesler S Akan M Iyilikci L Unek T Ocmen E et al. Comparison of the RIFLE, AKIN, and KDIGO diagnostic classifications for acute renal injury in patients undergoing liver transplantation. *Transplant Proc.* (2016) 48:2112–8. 10.1016/j.transproceed.2016.03.044 27569955

[36] Hilmi IA Damian D Al-Khafaji A Sakai T Donaldson J Winger DG et al. Acute kidney injury after orthotopic liver transplantation using living donor versus deceased donor grafts: a propensity score-matched analysis: acute kidney injury after liver transplantation. *Liver Transpl.* (2015) 21:1179–85. 10.1002/lt.24166 25980614 PMC4550550

[37] Hannon V Kothari RP Zhang L Bokoch MP Hill R Roll GR et al. The association between vena cava implantation technique and acute kidney injury after liver transplantation. *Transplantation.* (2020) 104:e308–16. 10.1097/TP.0000000000003331 32467477

[38] Haase M Devarajan P Haase-Fielitz A Bellomo R Cruz DN Wagener G et al. The outcome of neutrophil gelatinase-associated lipocalin (NGAL)-positive subclinical acute kidney injury: a multicenter pooled analysis of prospective studies. *J Am Coll Cardiol.* (2011) 57:1752–61. 10.1016/j.jacc.2010.11.051 21511111 PMC4866647

[39] Agarwal B. Difficulties in diagnosing acute kidney injury post liver transplantation using serum creatinine based diagnostic criteria. *World J Hepatol.* (2014) 6:696–703. 10.4254/wjh.v6.i10.696 25349641 PMC4209415

[40] Soh S Shim JK Song JW Kang B Kwak YL. Perioperative nadir hemoglobin concentration and outcome in off-pump coronary artery bypass surgery - a retrospective review. *Circ J.* (2020) 85:37–43. 10.1253/circj.CJ-20-0694 33229798

[41] Spolverato G Kim Y Ejaz A Frank SM Pawlik TM. Effect of relative decrease in blood hemoglobin concentrations on postoperative morbidity in patients who undergo major gastrointestinal surgery. *JAMA Surg.* (2015) 150:949–56. 10.1001/jamasurg.2015.1704 26222497

[42] Glance LG Dick AW Mukamel DB Fleming FJ Zollo RA Wissler R et al. Association between intraoperative blood transfusion and mortality and morbidity in patients undergoing noncardiac surgery. *Anesthesiology.* (2011) 114:283–92. 10.1097/ALN.0b013e3182054d06 21239971

[43] Vamvakas EC Blajchman MA. Transfusion-related mortality: the ongoing risks of allogeneic blood transfusion and the available strategies for their prevention. *Blood.* (2009) 113:3406–17. 10.1182/blood-2008-10-167643 19188662

[44] Hajjar LA Vincent JL Galas FRBG Nakamura RE Silva CMP Santos MH et al. Transfusion requirements after cardiac surgery: the TRACS randomized controlled trial. *JAMA.* (2010) 304:1559–67. 10.1001/jama.2010.1446 20940381

[45] Desai N Schofield N Richards T. Perioperative patient blood management to improve outcomes. *Anesth Analg.* (2018) 127:1211–20. 10.1213/ANE.0000000000002549 29064875

[46] Ellermann I Bueckmann A Eveslage M Buddendick H Latal T Niehoff D et al. Treating anemia in the preanesthesia assessment clinic: results of a retrospective evaluation. *Anesth Analg.* (2018) 127:1202–10. 10.1213/ANE.0000000000003583 29944518

[47] Keding V Zacharowski K Bechstein WO Meybohm P Schnitzbauer AA. Patient blood management improves outcome in oncologic surgery. *World J Surg Oncol.* (2018) 16:159. 10.1186/s12957-018-1456-9 30086770 PMC6081799

[48] Gupta PB DeMario VM Amin RM Gehrie EA Goel R Lee KHK et al. Patient blood management program improves blood use and clinical outcomes in orthopedic surgery. *Anesthesiology.* (2018) 129:1082–91. 10.1097/ALN.0000000000002397 30124488

[49] Froessler B Palm P Weber I Hodyl NA Singh R Murphy EM. The important role for intravenous iron in perioperative patient blood management in major abdominal surgery: a randomized controlled trial. *Ann Surg.* (2016) 264:41–6. 10.1097/SLA.0000000000001646 26817624 PMC4902320

[50] Stein J Connor S Virgin G Ong DE Pereyra L. Anemia and iron deficiency in gastrointestinal and liver conditions. *World J Gastroenterol.* (2016) 22:7908–25. 10.3748/wjg.v22.i35.7908 27672287 PMC5028806

[51] Mahamid M Mahroum N Bragazzi N Shalaata K Yavne Y Adawi M et al. Folate and B12 levels correlate with histological severity in NASH patients. *Nutrients.* (2018) 10:E440. 10.3390/nu10040440 29614799 PMC5946225

[52] Villanueva C Aracil C Colomo A Hernández-Gea V López-Balaguer JM Alvarez-Urturi C et al. Acute hemodynamic response to β-blockers and prediction of long-term outcome in primary prophylaxis of variceal bleeding. *Gastroenterology.* (2009) 137:119–28. 10.1053/j.gastro.2009.03.048 19344721

[53] Bihari C Anand L Rooge S Kumar D Saxena P Shubham S et al. Bone marrow stem cells and their niche components are adversely affected in advanced cirrhosis of the liver. *Hepatology.* (2016) 64:1273–88. 10.1002/hep.28754 27486864

