# Supplementary material for: Perioperative hemoglobin concentrations are associated with acute kidney injury after deceased donor liver transplantation
Source: Front Med (Lausanne). 2026 Mar 19;13:1689545. doi: 10.3389/fmed.2026.1689545 (PMC13044140; doi:10.3389/fmed.2026.1689545)
Supplement: Supplementary file 1 [file Data_Sheet_1.docx]

# Supplementary Tables

| **Supplementary Table 1. Etiology of liver disease.** | | | |
| --- | --- | --- | --- |
| *Etiology of liver disease* | *All Patients*  *n=724* | *No AKI*  *n=233* | *AKI*  *n=491* |
| Alcoholic liver cirrhosis | 207 (28.6) | 50 (21.5) | 157 (32) |
| Hepatocellular carcinoma | 177 (24.4) | 71 (30.5) | 106 (21.6) |
| Hepatitis C virus cirrhosis | 96 (13.3) | 30 (12.9) | 66 (13.4) |
| Sclerosing cholangitis | 41 (5.7) | 22 (9.4) | 19 (3.9) |
| Cryptogenic cirrhosis | 26 (3.6) | 8 (3.4) | 18 (3.7) |
| Acute on chronic hepatic failure | 24 (3.3) | 3 (1.3) | 21 (4.3) |
| Autoimmune cirrhosis | 19 (2.6) | 7 (3) | 12 (2.4) |
| Primary biliary cirrhosis | 18 (2.4) | 9 (3.9) | 9 (1.8) |
| Hepatitis B virus cirrhosis | 16 (2.2) | 1 (0.4) | 15 (3.1) |
| Wilson’s disease | 13 (1.8) | 3 (1.3) | 10 (2) |
| Non-alcoholic steatohepatitis | 12 (1.7) | 0 (0) | 12 (2.4) |
| Other | 75 (10.4) | 29 (12.4) | 46 (9.4) |
| Numbers are shown in n (%); Abbreviations: AKI, acute kidney injury. | | | |

| **Supplementary Table 2. Results of regression analyses on the association of postoperative hemoglobin concentrations with the indicated outcome after orthotopic liver transplantation** | | | | | | | | | | | |
| --- | --- | --- | --- | --- | --- | --- | --- | --- | --- | --- | --- |
|  | Univariable | | | Multivariable | | | | | | |  |
|  | OR/HR | 95% CI | P-value | OR | | 95% CI | | P-value | | |  |
| ***AKI (yes versus no) - logistic regression*** | | | | |  | |  | |  | | |
| Hb pre OP | 0.883 | 0.820-0.952 | **0.001** | 0.847 | | 0.773-0.928 | | **<0.001** | | |  |
| Hb POD 1 | 0.882 | 0.797-0.976 | **0.015** |  | |  | | n.s. | | |  |
| Hb POD 2^†^ | 0.878 | 0.767-1.004 | 0.058 |  | |  | |  | | |  |
| Hb nadir POD 1, 2^†^ | 0.746 | 0.644-0.865 | **<0.001** | 0.806 | | 0.661-0.983 | | **0.033** | | |  |
| Age | 1.014 | 0.999–1.030 | 0.065 |  | |  | |  | | |  |
| Gender (ref: F) | 1.824 | 1.295-2.570 | **0.001** | 1.921 | | 1.269-2.909 | | **0.002** | | |  |
| BMI | 1.114 | 1.071-1.158 | **<0.001** | 1.100 | | 1.050-1.152 | | **<0.001** | | |  |
| MELD | 1.043 | 1.019-1.068 | **<0.001** |  | |  | | n.s | | |  |
| AST POD 1 | 1.000 | 1.000-1.000 | **<0.001** | 1.000 | | 1.000-1.000 | | **<0.001** | | |  |
| ALT POD 1 | 1.000 | 1.000-1.001 | **<0.001** |  | |  | | n.s. | | |  |
| Bilirubin POD 1 | 1.069 | 1.025-1.115 | **0.002** |  | |  | | n.s | | |  |
| CRP POD 1 | 0.992 | 0.960-1.024 | 0.613 |  | |  | |  | | |  |
| eGFR | 1.002 | 0.996-1.007 | 0.539 |  | |  | |  | | |  |
| PRBCs intraoperative | 1.050 | 1.013-1.089 | **0.007** |  | |  | | n.s | | |  |
| FFP intraoperative | 1.036 | 1.013-1.059 | **0.002** |  | |  | | n.s | | |  |
| Warm ischemia time | 1.018 | 1.009-1.028 | **<0.001** |  | |  | | n.s | | |  |
| CHD (ref: no) | 1.217 | 0.501-2.957 | 0.664 |  | |  | |  | | |  |
| COPD (ref: no) | 1.091 | 0.558-2.133 | 0.800 |  | |  | |  | | |  |
| DM (ref: no) | 1.414 | 0.957-2.090 | 0.082 |  | |  | |  | | |  |
| ***AKI (0, 1, 2, 3) - ordered logistic regression*** | | | | |  | |  | |  | | |
| Hb pre OP | 0.876 | 0.822-0.932 | **<0.001** | 0.895 | | 0.826-0.970 | | **0.002** | | |  |
| Hb POD 1 | 0.898 | 0.822-0.980 | **0.016** |  | |  | | n.s. | | |  |
| Hb POD 2^†^ | 0.891 | 0.785-1.010 | 0.073 |  | |  | |  | | |  |
| Hb nadir POD 1, 2^†^ | 0.741 | 0.644-0.850 | **<0.001** | 0.782 | | 0.661-0.921 | | **<0.001** | | |  |
| Age | 1.014 | 1.001-1.028 | **0.040** | 1.021 | | 1.006-1.037 | | **0.003** | | |  |
| Gender (ref: F) | 1.357 | 0.999-1.848 | 0.051 |  | |  | |  | | |  |
| BMI | 1.091 | 1.058-1.124 | **<0.001** | 1.082 | | 1.046-1.119 | | **<0.001** | | |  |
| MELD | 1.053 | 1.033-1.074 | **<0.001** | 1.035 | | 1.010-1.060 | | **0.001** | | |  |
| AST POD 1 | 1.000 | 1.000-1.000 | **<0.001** | 1.000 | | 1.000-1.000 | | **<0.001** | | |  |
| ALT POD 1 | 1.000 | 1.000-1.001 | **<0.001** |  | |  | | n.s. | | |  |
| Bilirubin POD 1 | 1.054 | 1.022-1.089 | **0.001** |  | |  | | n.s | | |  |
| CRP POD 1 | 0.979 | 0.952-1.006 | 0.127 |  | |  | |  | | |  |
| eGFR | 0.997 | 0.992-1.002 | 0.243 |  | |  | |  | | |  |
| PRBCs intraoperative | 1.083 | 1.053-1.117 | **<0.001** | 1.047 | | 1.011-1.086 | | **0.011** | | |  |
| FFP intraoperative | 1.048 | 1.030-1.067 | **<0.001** |  | |  | | n.s. | | |  |
| Warm ischemia time | 1.016 | 1.009-1.025 | **<0.001** | 1.008 | | 1.000-1.016 | | **0.036** | | |  |
| CHD (ref: no) | 1.355 | 0.651-2.841 | 0.416 |  | |  | |  | | |  |
| COPD (ref: no) | 1.252 | 0.712-2.203 | 0.432 |  | |  | |  | | |  |
| DM (ref: no) | 1.232 | 0.902-1.684 | **0.191** |  | |  | | n.s. | | |  |
| Abbreviations: AKI, acute kidney injury; ALT, alanine aminotransferase; AST, aspartate aminotransferase; BMI, body mass index; CHD, coronary heart disease; CI, confidence interval; COPD, chronic obstructive pulmonary disease; CRP, c-reactive protein; DM, diabetes mellitus; eGFR, estimated glomerular filtration rate; F, female; FFP, fresh frozen plasma; Hb, hemoglobin concentrations; HR, hazard ratio; MELD, model for end-stage liver disease; n.s., not significant; OR, odds ratio; POD, postoperative day; PRBCs, packed red blood cells; pre OP, pre operative; ref, reference. † cohort with n = 522 excluding 202 patients with AKI>0 at POD 1 | | | | | | | | | |  |  |

| **Supplementary Table 3. Results of cox regression analyses on the association of perioperative parameters with time to acute kidney injury stage ≥ 1, stage ≥ 2, and stage 3 after orthotopic liver transplantation** | | | | | | | |  |
| --- | --- | --- | --- | --- | --- | --- | --- | --- |
|  | Univariable | | | Multivariable | | | |  |
|  | HR | 95% CI | P-value | | HR | 95% CI | P-value | |
| **A) Time to AKI stage ≥ 1** |  |  |  | |  |  |  | |
| *Hemoglobin concentrations* |  |  |  | |  |  |  | |
| Preoperative | 0.978 | 0.915-1.045 | 0.506 | |  |  |  | |
| Same day as AKI stage ≥ 1 | 0.980 | 0.880-1.092 | 0.716 | |  |  |  | |
| Day before AKI stage ≥ 1 | 1.040 | 0.966-1.120 | 0.294 | |  |  |  | |
| Δ same day to preoperative | 1.013 | 0.949-1.082 | 0.701 | |  |  |  | |
| Δ same day to day before | 0.950 | 0.882-1.024 | 0.179 | |  |  |  | |
| *Blood products* |  |  |  | |  |  |  | |
| PRBCs intraoperative | 1.024 | 1.004-1.045 | **0.019** | |  |  | n.s. | |
| PRBCs same day | 1.078 | 1.029-1.128 | **0.001** | | 1.088 | 1.018-1.162 | **0.013** | |
| FFP intraoperative | 1.026 | 1.011-1.041 | **0.001** | | 1.024 | 1.006-1.041 | **0.008** | |
| *Other perioperative parameters* | | |  | |  |  |  | |
| Age | 1.005 | 0.991-1.019 | 0.470 | |  |  |  | |
| Gender (ref: F) | 1.378 | 0.961-1.975 | 0.081 | |  |  |  | |
| BMI | 1.060 | 1.027-1.094 | **<0.001** | | 1.042 | 1.007-1.079 | **0.017** | |
| MELD | 1.031 | 1.010-1.052 | **0.003** | |  |  | n.s. | |
| CHD (ref: No) | 1.613 | 0.825-3.155 | 0.163 | |  |  |  | |
| COPD (ref: No) | 0.981 | 0.518-1.855 | 0.952 | |  |  |  | |
| DM (ref: No) | 1.532 | 1.114-2.105 | **0.009** | | 1.444 | 1.035-2.014 | **0.031** | |
| Bilirubin same day | 1.063 | 1.029-1.099 | **<0.001** | | 1.049 | 1.012-1.087 | **0.008** | |
| AST same day | 1.000 | 1.000-1.000 | **<0.001** | | 1.000 | 1.000-1.000 | **<0.001** | |
| ALT same day | 1.000 | 1.000-1.000 | **<0.001** | |  |  | n.s. | |
| CRP same day | 1.063 | 1.031-1.096 | **<0.001** | | 1.076 | 1.042-1.112 | **<0.001** | |
| eGFR | 1.001 | 0.996-1.006 | 0.658 | |  |  |  | |
| Warm ischemia time | 1.007 | 1.000–1.014 | 0.057 | |  |  |  | |
| **B) Time to AKI stage ≥ 2** |  |  |  | |  |  |  | |
| *Hemoglobin concentrations* |  |  |  | |  |  |  | |
| Preoperative | 0.936 | 0.861-1.017 | 0.119 | |  |  |  | |
| Same day as AKI stage ≥ 2 | 0.977 | 0.852-1.120 | 0.734 | |  |  |  | |
| Day before AKI stage ≥ 2 | 0.991 | 0.892-1.102 | 0.874 | |  |  |  | |
| Δ same day to preoperative | 1.053 | 0.970-1.142 | 0.215 | |  |  |  | |
| Δ same day to day before | 1.001 | 0.900-1.114 | 0.983 | |  |  |  | |
| *Blood products and albumin* |  |  |  | |  |  |  | |
| PRBCs intraoperative | 1.031 | 1.010-1.053 | **0.004** | |  |  | n.s. | |
| PRBCs same day | 1.100 | 1.044-1.159 | **<0.001** | | 1.098 | 1.038-1.162 | **0.001** | |
| FFP intraoperative | 1.031 | 1.014-1.048 | **<0.001** | |  |  | n.s. | |
| *Other perioperative parameters* | | |  | |  |  |  | |
| Age | 1.016 | 0.998-1.035 | 0.087 | |  |  |  | |
| Gender (ref: F) | 0.977 | 0.641-1.480 | 0.915 | |  |  |  | |
| BMI | 1.066 | 1.027-1.106 | **0.001** | | 1.068 | 1.028-1.110 | **0.001** | |
| MELD | 1.034 | 1.009-1.059 | **0.007** | |  |  | n.s. | |
| CHD (ref: No) | 1.936 | 0.902-4.157 | 0.090 | |  |  |  | |
| COPD (ref: No) | 0.995 | 0.464-2.135 | 0.990 | |  |  |  | |
| DM (ref: No) | 1.542 | 1.043-2.279 | **0.030** | |  |  | n.s. | |
| Bilirubin same day | 1.081 | 1.041-1.122 | **<0.001** | | 1.072 | 1.031-1.115 | **<0.001** | |
| AST same day | 1.000 | 1.000-1.000 | **<0.001** | | 1.000 | 1.000-1.000 | **<0.001** | |
| ALT same day | 1.000 | 1.000-1.000 | **<0.001** | |  |  | n.s. | |
| CRP same day | 1.077 | 1.039-1.117 | **<0.001** | | 1.086 | 1.049-1.124 | **<0.001** | |
| eGFR | 0.998 | 0.992-1.004 | 0.507 | |  |  |  | |
| Warm ischemia time | 1.008 | 0.999-1.017 | 0.083 | |  |  |  | |
| **C) Time to AKI stage 3** |  |  |  | |  |  |  | |
| *Hemoglobin concentrations* |  |  |  | |  |  |  | |
| Preoperative | 0.891 | 0.807-0.985 | **0.024** | |  |  | n.s. | |
| Same day as AKI stage 3 | 0.915 | 0.771-1.085 | 0.306 | |  |  |  | |
| Day before AKI stage 3 | 0.900 | 0.782-1.037 | 0.144 | |  |  |  | |
| Δ same day to preoperative | 1.079 | 0.979-1.189 | 0.124 | |  |  |  | |
| Δ same day to day before | 1.066 | 0.924-1.229 | 0.380 | |  |  |  | |
| *Blood products and albumin* |  |  |  | |  |  |  | |
| PRBCs intraoperative | 1.037 | 1.016-1.058 | **<0.001** | |  |  | n.s. | |
| PRBCs same day | 1.093 | 1.038-1.152 | **0.001** | | 1.090 | 1.027-1.157 | **0.005** | |
| FFP intraoperative | 1.035 | 1.015-1.055 | **<0.001** | |  |  | n.s. | |
| *Other perioperative parameters* | | |  | |  |  |  | |
| Age | 1.018 | 0.996-1.041 | 0.110 | |  |  |  | |
| Gender (ref: F) | 0.836 | 0.511-1.366 | 0.474 | |  |  |  | |
| BMI | 1.060 | 1.014-1.108 | **0.010** | | 1.063 | 1.014-1.113 | **0.010** | |
| MELD | 1.053 | 1.024-1.084 | **<0.001** | | 1.049 | 1.021-1.079 | **0.001** | |
| CHD (ref: No) | 2.289 | 0.997-5.252 | 0.051 | |  |  |  | |
| COPD (ref: No) | 0.711 | 0.260-1.939 | 0.505 | |  |  |  | |
| DM (ref: No) | 1.324 | 0.830-2.111 | 0.239 | |  |  |  | |
| Bilirubin same day | 1.081 | 1.039-1.125 | **<0.001** | |  |  | n.s. | |
| AST same day | 1.000 | 1.000-1.000 | **0.002** | |  |  | n.s. | |
| ALT same day | 1.000 | 1.000-1.000 | **0.002** | | 1.000 | 1.000-1.000 | **0.003** | |
| CRP same day | 1.013 | 0.960-1.069 | 0.644 | |  |  |  | |
| eGFR | 0.990 | 0.983-0.997 | **0.006** | |  |  | n.s. | |
| Warm ischemia time | 1.001 | 0.990-1.013 | 0.795 | |  |  |  | |

| Abbreviations: AKI, acute kidney injury; ALT, alanine aminotransferase; AST, aspartate aminotransferase; BMI, body mass index; CHD, coronary heart disease; CI, confidence interval; COPD, chronic obstructive pulmonary disease; CRP, c-reactive protein; Δ, difference; DM, diabetes mellitus; eGFR, estimated glomerular filtration rate; F, female; FFP, fresh frozen plasma; HR, hazard ratio; MELD, model for end-stage liver disease; n.s., not significant; PRBCs, packed red blood cells; ref, reference. |
| --- |
